# Generalized pustular psoriasis of the ‘von Zumbusch’-type: a sepsis mimic with low PCT values

**DOI:** 10.1007/s15010-024-02276-z

**Published:** 2024-05-02

**Authors:** Jörg C. Prinz, Lars E. French

**Affiliations:** Department of Dermatology and Allergy, University Hospital, Ludwig-Maximilian-University of Munich, Munich, Germany

**Keywords:** Sepsis mimics, Generalized pustular psoriasis

**Fig. 1:**
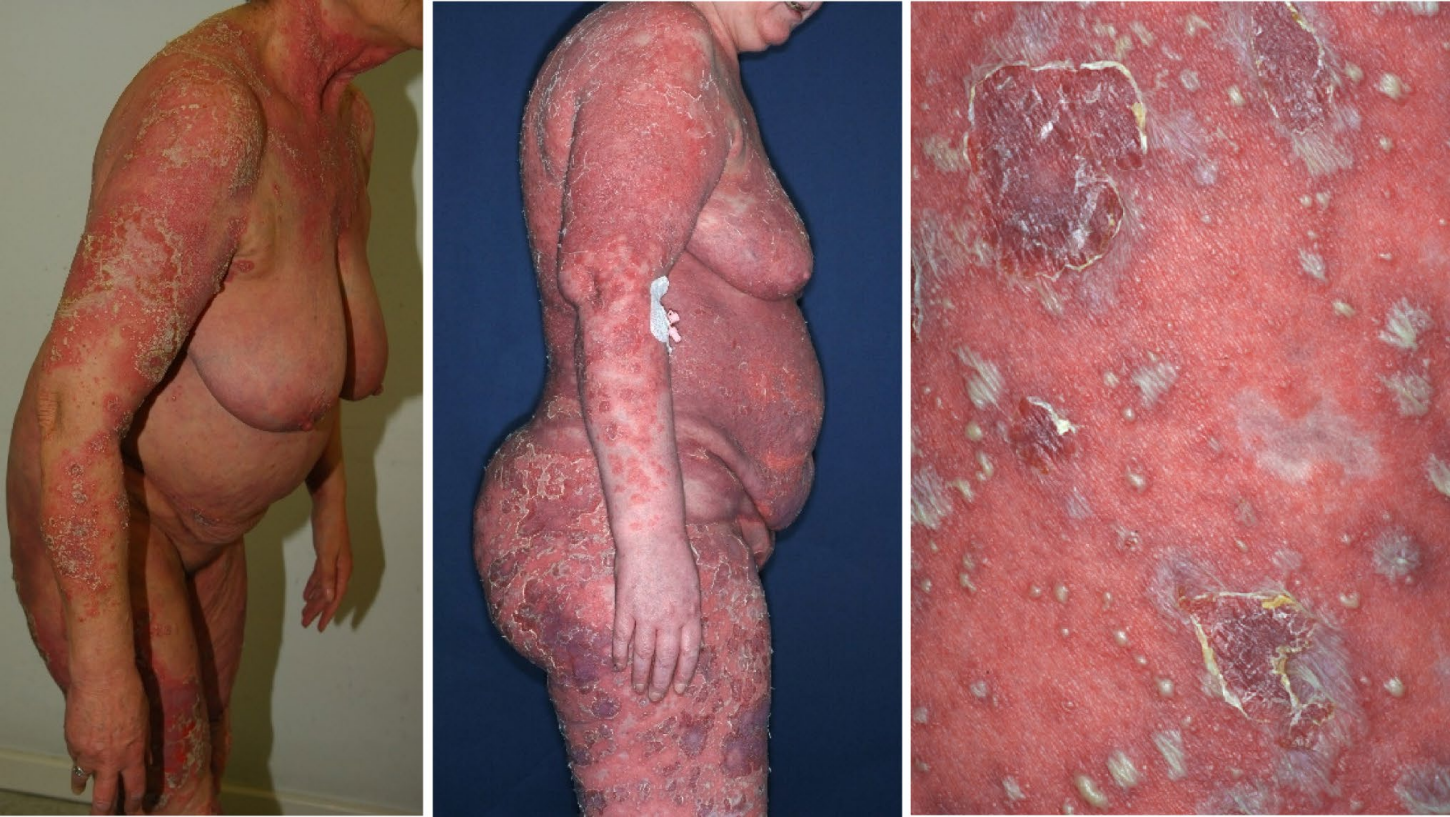
Patients with generalized pustular psoriasis of the ‘von Zumbusch’-type initially transferred to an ICU for presumed sepsis, and details of the pustular rash

The study "Sepsis mimics among presumed sepsis patients at intensive care admission: a retrospective observational study " by Maria Lengquist et al. [1] highlights the difficulty in the differential diagnosis between true sepsis and clinical conditions with sepsis-like symptoms at intensive care unit (ICU) admission, and it differentiates between various sepsis-like clinical presentations. Another important disease in the list of sepsis mimics is generalized pustular psoriasis of the ‘von-Zumbusch’-type. It is a rare inflammatory disease with autoinflammatory and autoimmune features, in which disturbances in the control of the proinflammatory activity of IL-36 due to mutations or functional insufficiency of *IL-36RN*, but also other proinflammatory predispositions such as mutations in *CARD14*, *MPO* and others [2], may suddenly lead to excessive systemic inflammatory episodes, either spontaneously or in association with psoriasis vulgaris. Patients with the ‘von Zumbusch’-type generalized pustular psoriasis develop severe malaise, extensive ‘shaking the bed’ shivering and high fever together with a generalized pustular rash (Fig. 1). If left untreated, the severe systemic inflammation can cause edematous swelling of the extremities, pleural effusions, renal failure and even exhaustion syndromes with lethal multi-organ failure [3]. The physical symptoms are accompanied by pronounced leukocytosis with predominant neutrophilia and high serum CRP levels. Due to the clinical symptoms including the pathological laboratory values and a pustular rash, which may be mistaken for a profuse bacterial skin infection, the disease is often misinterpreted as bacterial sepsis and patients are transferred to intensive care units for systemic therapy with antibiotics, glucocorticosteroids and even induction of artificial coma. An important criterion for distinguishing between GPP and sepsis is the procalcitonin (PCT) level in serum [4], which according to Maria Lengquist et al. [1] is often not routinely determined. PCT is upregulated in response to bacterial infections and has good discriminatory properties to differentiate between bacterial sepsis and the sepsis-like syndrome in generalized pustular psoriasis. PCT levels in the normal range can allow the diagnosis of generalized pustular psoriasis of the ‘von Zumbusch’-type in the presence of septic alterations and a pustular rash. Knowledge of the skin changes and the criteria differentiating generalized pustular psoriasis from bacterial sepsis and (Table 1) are therefore crucial for the correct diagnosis of generalized pustular psoriasis. A skin biopsy may further confirm the diagnosis through the histological detection of Kogoj's pustules with the accumulation of neutrophil granulocytes beneath the stratum corneum and thus exclude acute generalized exanthematous pustulosis, which is associated with eosinophilia in both blood and pustules. Treatment with cytokine antagonists neutralizing IL-36, TNF-α, IL-17, IL-12/23 or IL-23 can then rapidly improve the clinical condition [5].

**Table 1:** Criteria to distinguish generalized pustular psoriasis ‘von Zumbusch’-type from bacterial sepsis

Symptom	Generalized pustular psoriasis
Shivering	Yes
Fever	Yes
Generalized pustular rash	Yes
White blood cell counts	Highly elevated
Neutrophilia	Prominent
Serum CRP level	Highly elevated
Serum PCT level	Normal
History of psoriasis	Helpful
